# Application of Mean Modulus in Three-Point Bending and Roll Forming

**DOI:** 10.3390/ma16072571

**Published:** 2023-03-23

**Authors:** Menglong Xing, Haijun Wang, Jiyan Liu, Yutao Fu, Fengshan Du

**Affiliations:** 1 National Engineering Research Center for Equipment and Technology of Cold Strip Rolling, Yanshan University, Qinhuangdao 066004, China; 2 Qinhuangdao Chen-Ming Special-shaped Tube Manufacturing Co., Ltd., Qinhuangdao 066000, China

**Keywords:** mean modulus, finite element model, three-point bending tests, roll forming tests, springback prediction

## Abstract

Nonlinear unloading plays an important role in predicting springback during plastic forming process. To improve the accuracy of springback prediction which could provide a guide for precision forming, uniaxial tensile tests and uniaxial loading–unloading–loading tensile tests on SUS304 stainless steel were carried out. The flow stress mathematical model and chord modulus mathematical model were calibrated according to the test results. A constant elastic modulus three-point bending finite element model ($E_{0}FEMB$) and a constant elastic modulus roll forming finite element model ($E_{0}FEMR$) were established in MSC.MARC. The chord modulus was output by the PLOTV subroutine to determine the mean modulus of different regions, and the mean modulus three-point bending finite element model (${\bar{E}}_{c}FEMB$) and the mean modulus roll forming finite element model (${\bar{E}}_{c}FEMR$) were defined. The constant modulus finite element model ($E_{0}FEM$) simulation results and the mean modulus finite element model (${\bar{E}}_{c}FEM$) simulation results were compared with the three-point bending tests and roll forming tests test results. The difference between the simulation results and the test results was small, indicating that the mean modulus was feasible to predict the springback, which verified the suitability of the ${\bar{E}}_{c}FEM$.

## 1. Introduction

Bending parts account for a large proportion of mechanical parts, and springback is the key factor affecting the quality of them. The springback problem of materials generally exists in roll forming [1,2], stamping [3,4], bending [5,6] and other processes. Relative bending radius, bending angle and bending mode are the main factors affecting material springback. The influence of materials on springback cannot be ignored, such as titanium alloy, SUS304 stainless steel, etc. SUS304 stainless steel is widely used in aerospace, power machinery, petrochemical, biomedical [7] and other fields. The springback problem, which affects metal precision forming, will inevitably appear in the forming process of SUS304 stainless steel. With the increasing quality requirements of products, the influence of nonlinear unloading on springback has been paid more attention.

In related research of springback prediction, Sun [8] considered the influence of the Bauschinger effect and chord modulus variation on springback, and proposed the Quasi-Plastic-Elastic (QPE) model. Yoshida [9] proposed a model to determine the change in elastic modulus according to the current stress state, which effectively improved the calculation efficiency on the premise of satisfying the calculation accuracy. Yu [10] studied the change in chord modulus of TRIP steel in the nonlinear loading–unloading–loading, and determined that the change in chord modulus could predict the springback model. In addition, the prediction accuracy was improved by establishing a mixed hardening model to predict the springback of U-shaped parts. Chang [11] calibrated the chord modulus based on medium-Mn steel, and compared the constant elastic modulus and chord modulus through V bending tests under different working conditions; the validity of the chord modulus model was verified. Badr [12] studied the cycle hardening characteristics of Ti-6Al-4V alloy based on the homogenous yield function combined with the anisotropic hardening characteristics, and applied it to V bending and roll forming to predict the springback, which achieved good results. Chongthairungruang [13] conducted the s-rail stamping test of DP780 dual-phase steel, compared the test results with the simulation results, and the prediction accuracy of springback by the Yoshida-Uemori hardening model was higher. Yang [14] established an analytical model to predict the springback in Air-Bending of advanced high strength steel and updated it by a computer program based on classical bending theory.

In the related research on meshing in finite element, Nassiraei [15,16] a used sub-zone method to meshing different regions of FRP retrofitted X-connection, and meshing encryption for the extrapolation region. The simulation results were compared with the experimental data to verify the accuracy of the finite element model. Liu [17] took the pressure loss and heat transfer coefficient obtained by simulation as the targets to verify the grid independence of the Fin-and-flat tube heat exchangers, and determined the appropriate grid number considering the calculation efficiency and accuracy. The simulation results were compared with the experimental data to verify the correctness of the simulation model.

Based on the above research, it was seen that springback can be accurately predicted by considering the change in chord modulus in the unloading process. Therefore, it was necessary to analyze the change in chord modulus in the unloading process of SUS304 stainless steel and create a calculation model related to the chord modulus (mean modulus) to predict the springback during bending forming. At the same time, it was necessary to carry out mesh refinement and mesh sensitivity verification in the finite element model.

To predict springback accurately in three-point bending and roll forming, a calculation model of mean modulus was established based on the chord modulus mathematical model. In MSC.MARC (2013.1.0, MSC Software Corporation, Los Angeles, CA, USA), the ${\bar{E}}_{c}FEM$ was established based on the $E_{0}FEM$. The accuracy of the finite element model was verified by comparing the ${\bar{E}}_{c}FEM$ simulation results with the test results. The ${\bar{E}}_{c}FEM$ provides a reference for springback prediction.

## 2. Calibration of Flow Stress Mathematical Model and Chord Modulus Mathematical Model

### 2.1. Flow Stress Mathematical Model

**Figure 1:**
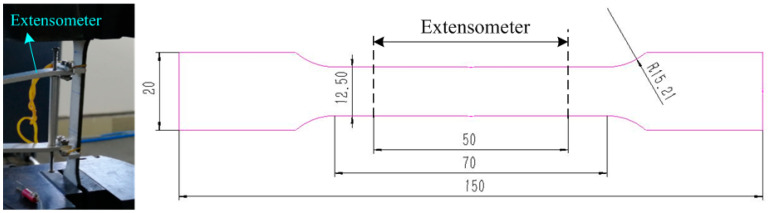
Tensile test and tensile sample.

Using SUS304 stainless steel sheet, the tensile sample in Figure 1 was processed by a wire electrical discharge machining. The chemical composition of the material is shown in Table 1. The sample thickness was 3 mm and the gauge length was 50 mm. The test machine crosshead speed was 3 mm/min. The surface of the tensile sample was polished with an 800-mesh paper. Tensile test at room temperature was carried out on the 100 kN electronic universal testing machine according to the China National Standard “Metallic materials-Tensile testing-Part 1: Method of test at room temperature” (GB/T 228.1-2010). When the sample reached the yield state, the extensometer was removed until the sample broke. In the test process, the force sensor and displacement sensor converted the force signal and displacement signal into an electrical signal. The two signals were digitized after A/D conversion, and then input to the computer via the interface circuit. The received digital quantity was converted into analog quantity by D/A conversion through the computer, and finally the stress–strain curve was drawn. The test process is shown in Figure 1.

**Figure 2:**
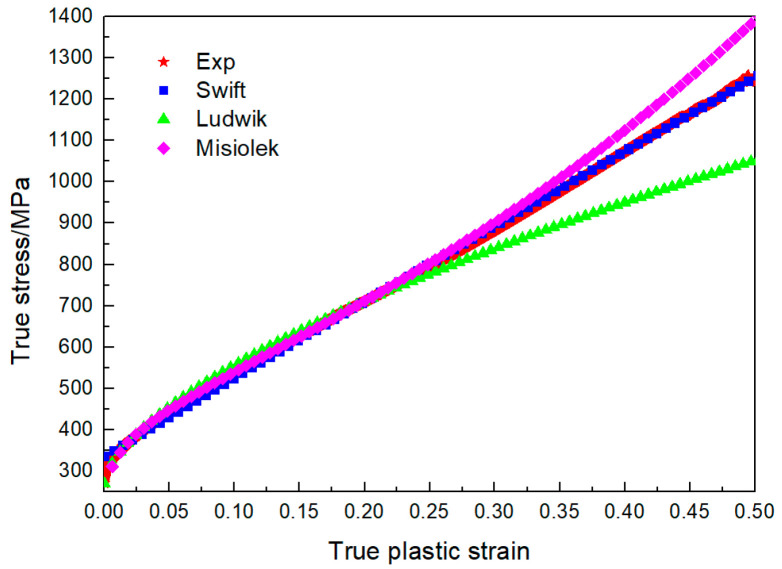
Fitting results of different flow stress mathematical models.

Flow stress data at the plastic stage were fitted based on the isotropic hardening model, as shown in Figure 2. The three groups of test data selected for Swift model calibration were (0.02, 375), (0.2, 711.14) and (0.4, 1074.29). The three groups of test data selected for Ludwik model calibration were (0, 270.61), (0.02, 375) and (0.2, 711.14). The three groups of test data selected for Misiolek model calibration were (0.02, 375), (0.1, 539.6), and (0.2, 711.14). The calibration results are shown in Table 2, and the Swift model had the best fitting, so the Swift model was selected as the material model in the plastic stage.

### 2.2. Chord Modulus Mathematical Model

**Figure 3:**
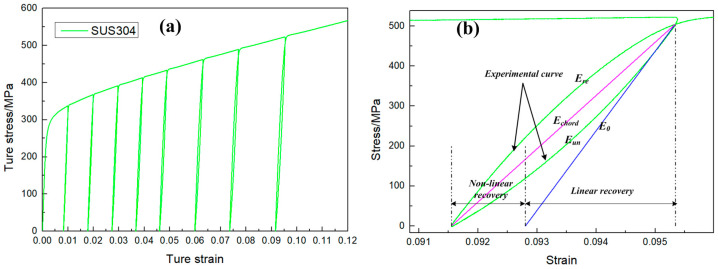
Results of uniaxial loading–unloading–loading tensile test. (**a**) The testing curve (**b**) Display of the chord modulus.

The tensile sample in Figure 1 was used for an uniaxial loading–unloading–loading tensile test. The test process was the following: the tensile sample was loaded to a pre-strain, then the loading was stopped and the stress unloaded to zero; finally, the sample was reloaded to the next pre-strain. The pre-strain was set as 1%, 2%, 3%, 4%, 5%, 6.5%, 8% and 10%. The chord modulus changed under different strains, and the upper yield limit increased during reloading (Figure 3a). Figure 3b shows the unloading–loading curve with $\varepsilon_{p}=0.1$, and the recovery stage was divided into linear recovery and nonlinear recovery. The slope of the chord modulus was different from the initial modulus, and there was an obvious decrease in the chord modulus. The resulting phenomenon was generally believed to be related with dislocation behavior [21].

The chord modulus mathematical model proposed by Yoshida [22] was used to fit the test data in Figure 3:

(1) $$E_{c}=E_{0}-\left(E_{0}-E_{a}\right)\cdot \left[1-\exp\left(-\xi \cdot {\bar{\varepsilon }}_{p}\right)\right],$$

where $E_{c}\left(MPa\right)$ is the chord modulus, $E_{0}\;\left(MPa\right)$ is the initial elastic modulus, $E_{a}\left(MPa\right)$ is the saturated elastic modulus, ${\bar{\varepsilon }}_{p}$ is the equivalent plastic strain, and ξ is the material constant.

**Figure 4:**
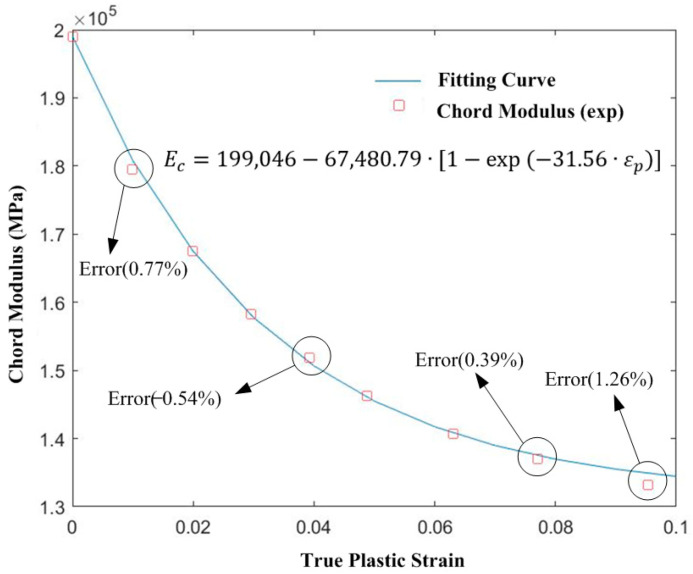
Fitting of the chord modulus mathematical model.

The three groups of test data selected for chord modulus mathematical model calibration were (0, 199,046), (0.02, 167,461.84) and (0.08, 136,968.7). Figure 4 shows that when the equivalent plastic strain was small, the chord modulus decreased rapidly, and with increased strain, the decreasing rate of chord modulus slowed down gradually and tended to be stable. When the true plastic strain was 0.0953, the error between the test and the model reached a maximum of 1.26%, which was still within the acceptable range (less than 5%). The calibrated model showed the change in chord modulus.

## 3. Finite Element Model

### 3.1. Establishment of the Finite Element Model

**Figure 5:**
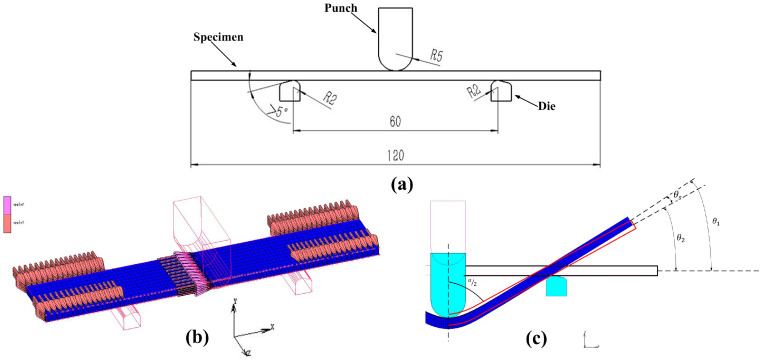
Finite element model of the three-point bending. (**a**) Parameters of three-point bending test; (**b**) Finite element model; (**c**) Evaluation index of springback calculation.

In MSC.MARC, a three-point bending finite element model (Figure 5b) was established based on Figure 5a. The Von MISES yield criterion was adopted by the model. The friction coefficient between the specimen and the support die was set at 0.2, and that between the punch and the specimen was set at 0. Element 7 was used for grid division. The punch was controlled to move downward through the TABLE method. The displacement constraint in the z direction was applied to the end of the specimen, and the displacement constraint in the x direction was applied to the center of the specimen (Figure 5b). The elastic modulus was 199,046 (MPa) and Poisson’s ratio was 0.3. The evaluation index of springback calculation (α) in three-point bending is shown in Figure 5c.

**Figure 6:**
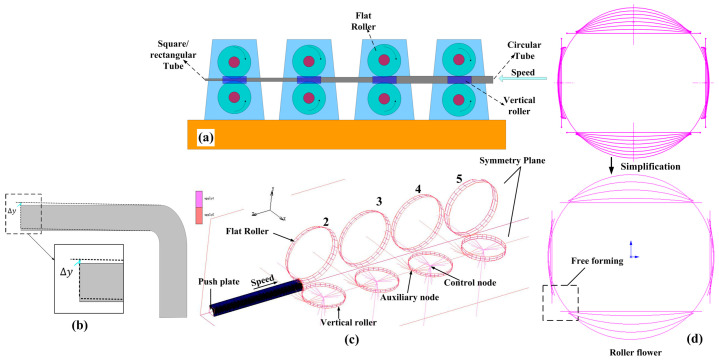
Finite element model of the roll forming. (**a**) Schematic diagram of rolling forming; (**b**) Evaluation index of springback calculation; (**c**) Finite element model; (**d**) Roller flower.

The process of circular tube roll forming rectangular tube is shown in Figure 6a. In the actual production process, the configuration relationship between passes should meet the bite condition [23]. However, in order to improve the computational efficiency in the simulation process, the model was simplified by reducing the number of passes (Bite conditions and stand spacing were ignored) (Figure 6), and reasonable pass allocation has little influence on springback. According to the roller flower (Figure 6d), the 1/4 finite element model was used for analysis in MSC.MARC. Five passes were used in the forming process: the first pass was the sizing stage, and the model was established from the second pass. Two planes of symmetry were established in the x and y directions (Figure 6c). The Von MISES yield criterion was adopted in the model, and the friction coefficient between the flat roller and tube was 0.2. The friction coefficient between the vertical roller and tube was 0.001. Element 7 was used for grid division, and mesh refinement was carried out on the corner forming area. The speed of the pushing plate had to be slightly lower than the linear speed of the entrance roller at the entrance of the forming pass. Control node and auxiliary node were used to constrain the vertical roller. The roller diameter increases gradually with the increase in the number of passes according to the principle of equal metal flow per second. The finite element model is shown in Figure 6c, and the process parameters in roll forming are shown in Table 3. The evaluation index of springback (Δy) calculation in roll forming is shown in Figure 6b.

### 3.2. Analysis of Simulation Results

**Figure 7:**
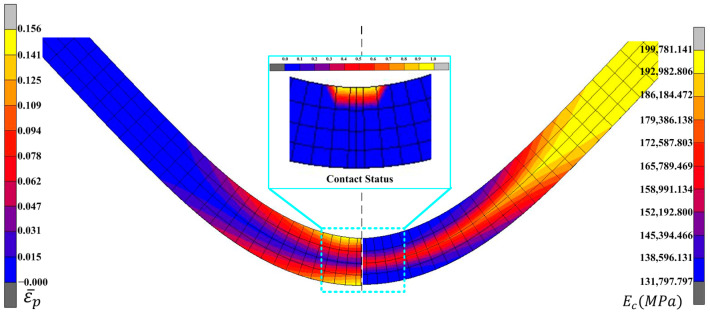
Simulation results of the equivalent plastic strain and chord modulus in three point-bending.

In MSC.MARC, the chord modulus was output by the PLOTV subroutine based on the chord modulus mathematical model. The simulation results of equivalent plastic strain and chord modulus before springback are shown in Figure 7, which shows that the chord modulus gradually decreased with increased equivalent plastic strain.

**Figure 8:**
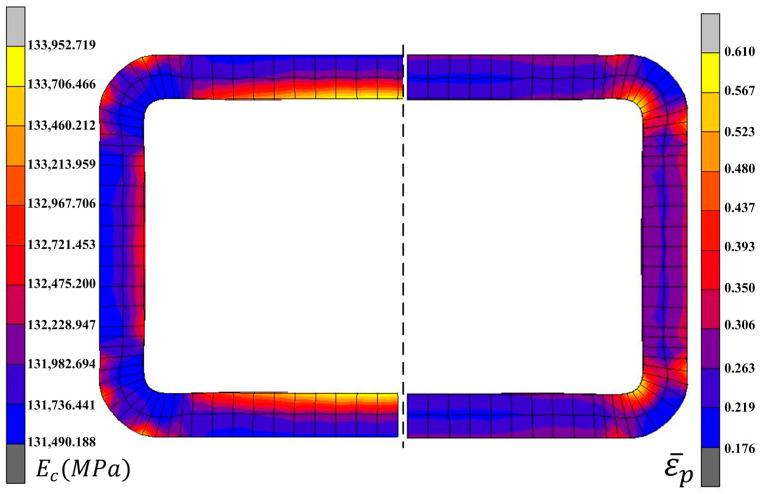
Simulation results of the chord modulus in roll forming.

The chord modulus distribution of the final forming pass in roll forming is shown in Figure 8. The chord modulus on the inner region of the corner was small, which matched the equivalent plastic strain distribution in the forming process.

**Figure 9:**
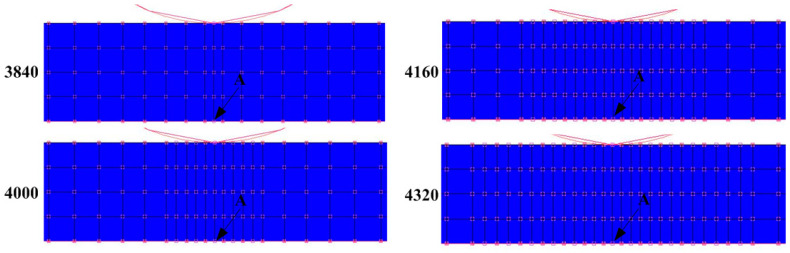
Different division schemes of mesh refinement.

**Figure 10:**
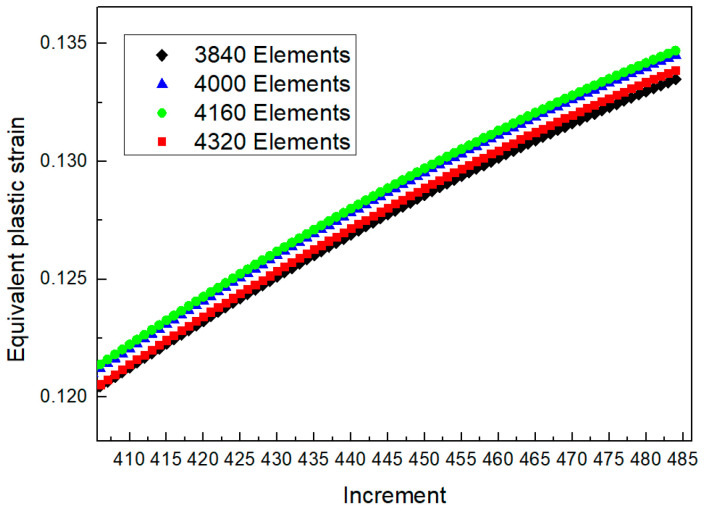
Analysis of grid sensitivity.

It was necessary to analyze the grid sensitivity of the finite element model to obtain more accurate finite element calculation results. Taking the grid division of the sheet as an example, the mesh of the main deformation region was refined (Figure 9). Four meshing methods were adopted to generate 3840, 4000, 4160, and 4320 elements, respectively. The equivalent plastic strain increment curves on point A (Figure 9) of the above four meshing methods are shown in Figure 10. The change in the element number was no longer sensitive to the forming result.

### 3.3. Setting of the Mean Modulus

To improve the prediction accuracy of springback, the average values of the initial elastic modulus ($E_{0}$) and the chord modulus ($E_{c}$) were calculated as the mean modulus of different regions. The definition of the mean modulus is

(2) $${\bar{E}}_{i}=\frac{\left(E_{0}+E_{c}\right)}{2},$$

where ${\bar{E}}_{i}\;\left(i=1,2\ldots n\right)$ is the mean modulus, which was defined as a constant in the simulation model. By setting the mean modulus of the initial specimen and tube, the ${\bar{E}}_{c}FEM$ was established.

**Figure 11:**
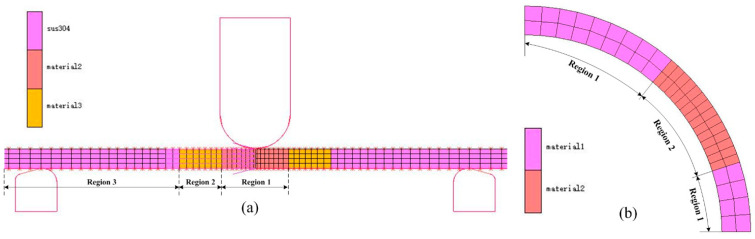
Setting of the mean modulus. (**a**) Three-point bending; (**b**) Roll forming.

The specimen was divided into three regions for mean modulus assignment (Figure 11a) according to the chord modulus distribution in Figure 7. The circular tube was divided into the corner region and the non-corner region for mean modulus assignment (Figure 11b) based on the chord modulus distribution in Figure 8. The mean modulus values of different regions in the finite element model are shown in Table 4.

## 4. Test Verification of the Mean Modulus Model

### 4.1. Three-Point Bending Test

**Figure 12:**
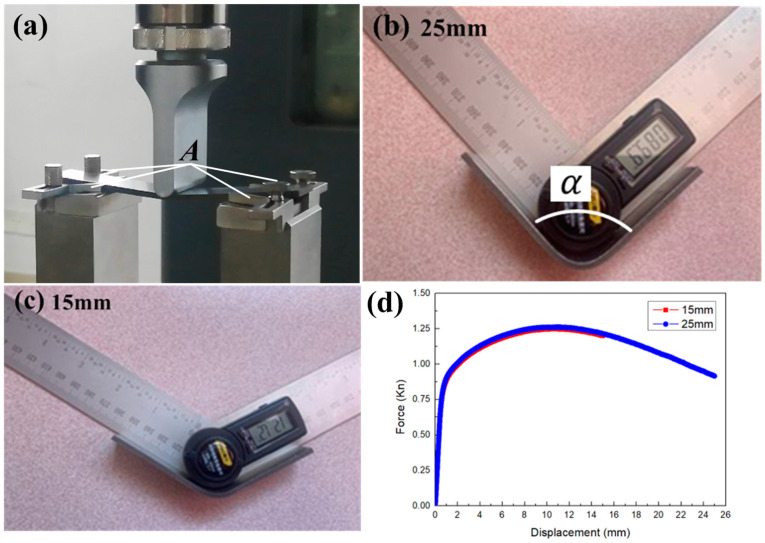
Three-point bending test results. (**a**) Test; (**b**) The forming result at the displacement of 25 mm; (**c**) The forming result at the displacement of 15 mm; (**d**) Testing curves.

To verify the accuracy of the mean modulus, the three-point bending test was carried out according to the China National Standard “Metallic materials-Determination of bending mechanical properties” (GB/T 14452-1993). The three-point bending test device was composed of a punch and two supporting dies. Two clamps were fixed on each side of the end of the specimen (Figure 12a A) to prevent the specimen from moving back and forth and causing instability. The length of the specimen was 120 mm; the width was 20 mm; the radius of the punch was 5 mm; the radius of the die was 2 mm; and the span was 60 mm. The punch moved down 15 mm and 25 mm. The forming results are shown in Figure 12b,c. When the displacement was 25 mm, the α of the sheet was 89.9°. When the displacement was 15 mm, the α of the sheet was 121.2°. Both conditions had reached the maximum load (Figure 12d—testing curves) and the plastic deformation was sufficient, which could be used to verify the mean modulus.

**Figure 13:**
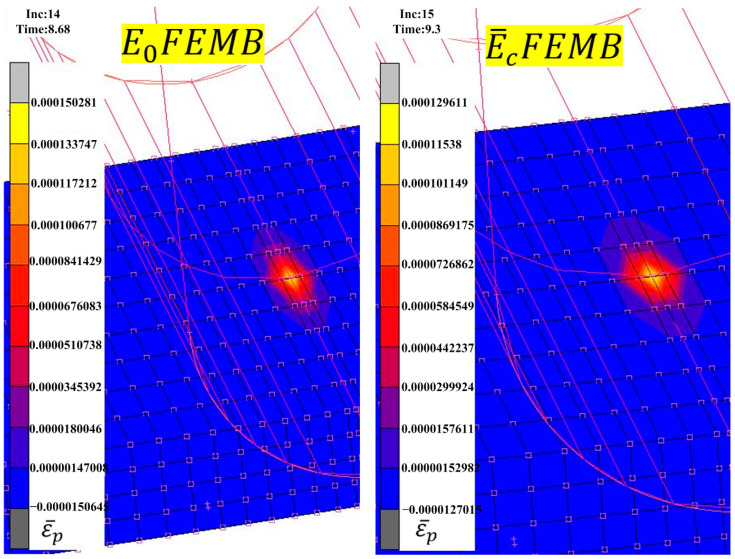
Comparison of equivalent plastic strain at the beginning of the plastic stage.

By comparing the $E_{0}FEMB$ simulation results and the ${\bar{E}}_{c}FEMB$ simulation results with the test results (Table 5), the difference between the ${\bar{E}}_{c}FEMB$ and the test results was small. When the displacement was 15 mm, the error between the three was less than 1%. At 25 mm, the prediction accuracy of the ${\bar{E}}_{c}FEMB$ increased by 1.22% compared with that of the $E_{0}FEMB$. The prediction results of the ${\bar{E}}_{c}FEMB$ are more consistent with the test results (Table 5), which verified the effectiveness of the method. When α was used as the evaluation index, the simulation result of ${\bar{E}}_{c}FEMB$ was smaller than that of $E_{0}FEMB$ (Table 5). The reason for this was that different chord modulus would change when the model reached the plastic stage. The model would enter the plastic stage later when the chord modulus was low (Figure 13). Therefore, a lower flow stress of ${\bar{E}}_{c}FEMB$, provided a smaller α value under the same displacement condition.

### 4.2. Roll Forming Test

**Figure 14:**
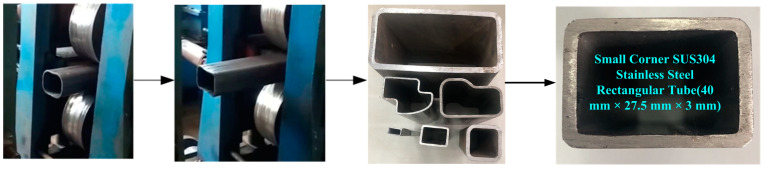
Roll forming test of shaped tube.

In roll forming, springback was a common problem in sheet metal forming (Open forming) [24], but the springback problem of circular tube roll forming rectangular tube (Closed forming) cannot be ignored. The forming experiment of SUS304 stainless steel rectangular tube (40 × 27.5 × 3 mm) with a small corner was studied by multi-pass method as shown in Figure 14. Roll forming was finished by two flat rollers that transmited power through the gears, and the power of the gearbox was provided by a motor.

**Figure 15:**
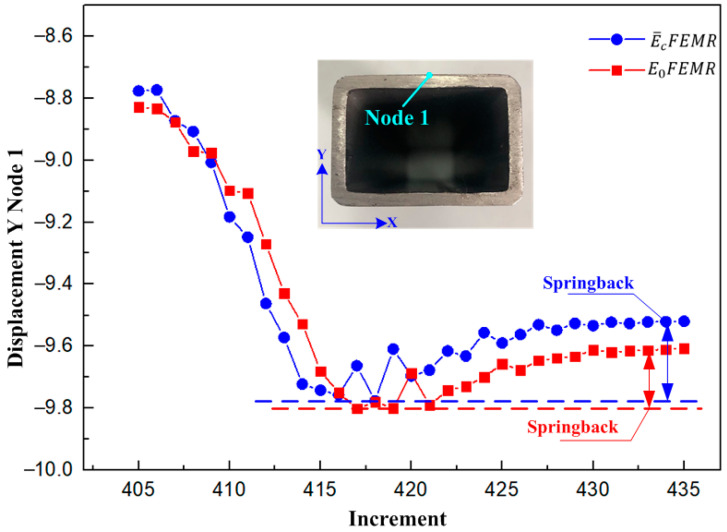
Comparison of springback simulation results of the $E_{0}FEMR$ and the ${\bar{E}}_{c}FEMR$.

The springback result of the ${\bar{E}}_{c}FEMR$ was larger than that of the $E_{0}FEMR$ according to the Node 1 displacement on the Y direction (Figure 15). The error between the ${\bar{E}}_{c}FEMR$ results and the test results was 15% according to the springback distance on the Y direction (Table 6). The ${\bar{E}}_{c}FEMR$ results are closer to the test results, which verified the effectiveness of the ${\bar{E}}_{c}FEMR$.

## 5. Conclusions

(1) For SUS304 stainless steel, the flow stress mathematical model and chord modulus mathematical model were calibrated by uniaxial tensile tests and loading–unloading–loading tensile tests. The two mathematical models fitted well with the test results and could be used for finite element simulation.
(2) Constant modulus finite element models for three-point bending and roll forming were established. The chord modulus distribution was output by the PLOTV subroutine in MSC.MARC, and the mean modulus was calculated based on this. The mean modulus was set for different forming regions, and the ${\bar{E}}_{c}FEM$ based on the mean modulus was established.
(3) Combined with the three-point bending tests and the roll forming tests, the simulation results of $E_{0}FEM$ and ${\bar{E}}_{c}FEM$ were compared with the test results. In three-point bending forming, when the displacement was 15 mm, the error between the ${\bar{E}}_{c}FEMB$-calculated results and the test results was 0.84%. When the displacement was 25 mm, the error between the ${\bar{E}}_{c}FEMB$-calculated results and the test results was 1.16%. In roll forming, the error between the ${\bar{E}}_{c}FEMR$-calculated results and the test results was 15%, which was within an acceptable range (less than 20%) and verified the validity of the ${\bar{E}}_{c}FEM$.

## Figures and Tables

**Table 1:** Standard chemical composition of SUS304 stainless steel (wt%).

C	Si	Mn	P	S	Ni	Cr	Fe
≤0.08	≤1	≤2	≤0.045	≤0.03	8~10.5	18~20	Remaining

**Table 2:** Calibration results of different flow stress mathematical models.

Model	Material Parameters	Correlation Coefficient
Swift [18]: $\sigma =A_{1}{\left(\varepsilon_{p}+\varepsilon_{0}\right)}^{n_{1}}$	$A_{1}=1840.41438,\varepsilon_{0}=0.1665$ $n_{1}=0.947$	0.99996
Ludwik [19]: $\sigma =\sigma_{s}+A_{2}\varepsilon_{p}{}^{n_{2}}$	$\sigma_{s}=270.61,A_{2}=1205.1831$ $n_{2}=0.6253$	0.98873
Misiolek [20]: $\sigma =A_{3}\varepsilon_{p}{}^{n_{3}}\cdot \exp\left(b\varepsilon_{p}\right)$	$A_{3}=614.633,n_{3}=0.1356$ b=1.821	0.99204

σ is the flow stress; $A_{1},\sigma ,\varepsilon_{0},n_{1},\sigma_{s},A_{2},n_{2},A_{3},n_{3}$ and b are material constants.

**Table 3:** Process parameters of the roll forming.

Parameters	Friction Coefficient of Flat Roller	Friction Coefficient of Vertical Roller	Speed of Flat Roller (rad/s)	Diameter of the Circular Tube (mm)	Thickness of the Circular Tube (mm)	Diameter of the Second Pass at the Top of the Roller (mm)	Roller Diameter of the 5th Pass (mm)
values	0.2	0.001	2	47	3	158	164

**Table 4:** Mean modulus setting in different regions.

Different Regions	Region 1 (MPa)	Region 2 (MPa)	Region 3 (MPa)
${\bar{E}}_{C}\;\left(15\;\mathrm{mm}\right)$	177,772	182,634	199,046
${\bar{E}}_{C}\;\left(25\;\mathrm{mm}\right)$	177,595	181,212	199,046
${\bar{E}}_{C}\;\left(roll\;forming\right)$	165,000	150,000	-

**Table 5:** Comparison of springback results in three-point bending.

$\alpha^{{}^{\circ}}$	$E_{0}FEMB$	${\bar{E}}_{c}FEMB$	Test Results	The Error Between $E_{0}FEMB$ and Test Results	The Error Between ${\bar{E}}_{c}FEMB$ and Test Results
15 mm	122.26	122.22	121.2	0.87%	0.84%
25 mm	92.04	90.94	89.9	2.38%	1.16%

**Table 6:** Comparison of springback results in roll forming.

Δy (mm)	$E_{0}FEMR$	${\bar{E}}_{c}FEMR$	Test Results	Error Between $E_{0}FEMR$ and Test Results	Error Between ${\bar{E}}_{c}FEMR$ and Test Results
	0.14	0.23	0.2	−30%	15%

## Data Availability

Not applicable.
